# Rosiglitazone inhibits metastasis development of a murine mammary tumor cell line LMM3

**DOI:** 10.1186/1471-2407-8-47

**Published:** 2008-02-08

**Authors:** Gabriela Magenta, Ximena Borenstein, Romina Rolando, María Adela Jasnis

**Affiliations:** 1 Research Area, Institute of Oncology AH Roffo. Av. San Martín 5481 (CP 1417). Buenos Aires, Argentina

## Abstract

**Background:**

Activation of peroxisome proliferator-activated receptors γ (PPARγ) induces diverse effects on cancer cells. The thiazolidinediones (TZDs), such as troglitazone and ciglitazone, are PPARγ agonists exhibiting antitumor activities; however, the underlying mechanism remains inconclusive. Rosiglitazone (RGZ), a synthetic ligand of PPARγ used in the treatment of Type 2 diabetes, inhibits growth of some tumor cells and is involved in other processes related to cancer progression. Opposing results have also been reported with different ligands on tumor cells. The purpose of this study was to determine if RGZ and 15d-PGJ_2 _induce antitumor effects *in vivo *and *in vitro *on the murine mammary tumor cell line LMM3.

**Methods:**

The effect on LMM3 cell viability and nitric oxide (NO) production of different doses of RGZ, 15-dPGJ_2_, BADGE and GW9662 were determined using the MTS colorimetric assay and the Griess reaction respectively. *In vivo *effect of orally administration of RGZ on tumor progression was evaluated either on s.c. primary tumors as well as on experimental metastasis. Cell adhesion, migration (wound assay) and invasion in Transwells were performed. Metalloproteinase activity (MMP) was determined by zymography in conditioned media from RGZ treated tumor cells. PPARγ expression was detected by inmunohistochemistry in formalin fixed tumors and by western blot in tumor cell lysates.

**Results:**

RGZ orally administered to tumor-bearing mice decreased the number of experimental lung metastases without affecting primary s.c. tumor growth. Tumor cell adhesion and migration, as well as metalloproteinase MMP-9 activity, decreased in the presence of 1 μM RGZ (non-cytotoxic dose). RGZ induced PPARγ protein expression in LMM3 tumors. Although metabolic activity -measured by MTS assay- diminished with 1–100 μM RGZ, 1 μM-treated cells recovered their proliferating capacity while 100 μM treated cells died. The PPARγ antagonist Biphenol A diglicydyl ether (BADGE) did not affect RGZ activity. On the contrary, the specific antagonist GW9662 completely abrogated RGZ-induced decrease in cell viability. A decrease in NO levels was detected in the presence of either 1 or 100 μM RGZ. The natural ligand 15d-PGJ_2 _did not affect metabolic activity although it induced a significant decrease in NO production.

**Conclusion:**

A significant decrease in the number of experimental LMM3 lung metastasis, but not on primary tumor growth, after oral RGZ administration was observed. *In vitro*, 100 μMRGZ also reduced cell viability and NO production, while no changes were observed in the presence of 15d-PGJ_2_. BADGE did not reverse RGZ effect while the antagonist GW9662 completely abrogated it, suggesting a PPARγ- dependent mechanism. Inhibition of lung metastatic nodules by RGZ administered in vivo, might be associated with the observed decrease in MMP-9 expression, in cell adhesion, migration and invasion. RGZ augmented its expression. PPARγ was detected in cell lysates by western blot and by immunohistochemistry in tumors from RGZ-treated mice. In summary we can suggest that RGZ or any other TZDs might be possible future approaches in the treatment of metastasis of PPARγ-expressing cells.

## Background

Peroxisome proliferator activated receptors (PPARs) belong to a ligand-dependent nuclear receptor family that regulates multiple metabolic processes associated with β-oxidation, glucose utilization and cholesterol transport. Three subtypes of PPARs (α, β/δ and γ) have been identified exhibiting distinct tissue distribution and associated with selective ligands [1]. PPARγ is broadly characterized and it is highly expressed in adipose and adrenal tissues, colon epithelia, T and B lymphocytes and macrophages.

The heterodimer PPARγ-RXR (retinoic acid receptor) binds specifically to response elements and works as a transcription factor [2]. Because of the recruitment of different coactivators PPARγ shows distinctive biological activities [3]. As seen in monocytes and macrophages, ligands can repress the transcription of proinflammatory products such as TNF, IL-1 and iNOS (inducible nitric oxide synthase) [4,5].

The role of PPARs is controversial in tumor biology. PPARγ ligands have anticancer effects against a wide variety of neoplastic cells *in vitro *and *in vivo *but target genes involved in this activity remain unclear [6]. Many authors have documented induction of differentiation and apoptosis by ligands of PPARγ, particularly in non-small cell lung cancer, glioblastoma, prostate, colon, pituitary and liver cancer cells [6-8]. Invasion of breast cancer cells, that often express prominent levels of PPARγ, is inhibited by rosiglitazone (RGZ) in a PPARγ-independent manner [9].

Since we first found that murine mammary LMM3 tumor cells expressed PPARγ protein, we performed *in vivo *experiments to analyze the effect of RGZ, the synthetic compound with high affinity for PPARγ, on primary tumor and lung metastasis development. Furthermore, we studied the effects of RGZ on tumor cell viability, adhesion, migration and levels of MMP-9 and NO production. Activity of the natural ligand 15-deoxy-Δ^12,14^-PGJ_2 _(15d-PGJ_2_) was studied. Experiments with the PPARγ antagonist bisphenol A diglycidyl ether (BADGE) [10] and GW 9662 [11] were included to evaluate the roles of PPARγ-dependent and independent signaling pathways in several responses. According to our results, RGZ might be considered as a possible therapeutic, able to inhibit tumor cell growth in secondary tissues affecting different steps of the metastatic cascade. The involvement of the inflammatory milieu associated with tumor development must be also considered.

## Methods

### Reagents

15-deoxy-delta^12,14^-prostaglandin J_2 _(15d-PGJ_2_, Cayman Chemical Company, Ann Arbor, MI, USA) and Rosiglitazone -RGZ- (ELEA Lab, Argentina) were dissolved in 100% and 70% ethanol respectively. Bisphenol A diglycidyl ether (BADGE, FLUKA, Chemical GmbH Buchs, Switzerland), was dissolved in 70% ethanol and GW9662 (Sigma-Aldrich, USA) in DMSO. Each of the solvents was used as a control without significant effect. LPS (Sigma- Aldrich, Missouri, USA.) was used at 500 μg/ml.

### Tumor cell culture

The tumor cell line LMM3, syngeneic to BALB/c, derived from spontaneous lung metastasis from the primary MM3 mammary tumor, was obtained and established in our Research Area [12]. LMM3 cells were maintained in MEM medium (Gibco, BRL) with 3 mM L-glutamine, 80 μM/ml gentamycin, supplemented with 5% fetal calf serum (FCS) (Bioser, Argentina) and cultured in a humidified 5% CO_2 _air atmosphere at 37°C. Serial passages of confluent monolayers were performed by detaching cells with trypsin (0.25% trypsin and 0.075% EDTA in CA^++ ^and Mg^++ ^free PBS). Medium was replaced every two days. LMM3 cells were used for *in vitro *and *in vivo *studies.

### Cell viability

1 × 10^4^ cells/200 μL MEM + 10% FCS were seeded in 96-well plates (CELLSTAR, Greiner Bio-One, USA). After 2 h adhesion, cells were washed and fresh medium with RGZ or 15d-PGJ2 (0.01–100 uM) without FCS was added. When used, BADGE (10 μM) was added 15 min and GW-9662 (1, 10, 20 μM) 1 h before RGZ. After 24 h incubation, metabolic activity was measured using the MTS assay (CellTiter96 AQueous Non-radioactive Cell Proliferation Assay, Promega, USA) an ELISA plate reader (Bio Rad) at 492 nm wavelength. In addition, cells were cultured for 24–48 h with 1–100 uM RGZ and then placed back in fresh medium for additional 48 h. Number of viable cells was quantified by Trypan blue exclusion test every 24 h up to 96 h.

### NO production

The level of NO was estimated by measuring nitrite levels with Griess reagent in extracellular medium from cells incubated overnight with RGZ and 15d-PGJ_2_. Briefly, 100 μl cell-free supernatants were added to equal volume of Griess reagent [13] (1% sulphanyl amine in 30% acetic acid with 0.1 % N-1-naphtyl ethylendiamine dihydrochloride in 60 % acetic acid). Absorbance was measured at 540 nm with an ELISA reader. Different concentrations of sodium nitrite were used to construct a standard curve.

### In vivo tumor growth

At day 0, female inbred BALB/c mice (aged 6–8 weeks) were s.c. inoculated into the flank with LMM3 cells (3 × 10^5^/0.1 ml). After inoculation, mice were randomized into 2 groups (each with n= 10). One group was administered a concentration of 100 μM RGZ in the drinking water while control group was administered with ethanol 0.01 % v/v. Treatment continued for 30 days (total duration of the experiment). Animals were scored for tumor growth three times/week by measuring the smallest and largest diameters of the s.c. tumors with a Vernier caliper. At the end of the experiment, some tumors were excised to determine PPARγ expression. Mice were maintained under guidelines established in the guide for Care and Use of Laboratory Animals (NIH publications, 1986).

### Experimental metastasis in vivo

2 × 10^5 ^LMM3 cells were i.v. injected into the tail vein. RGZ (100 uM) was administered in the drinking water. Ethanol was administered to control mice. At day 30 mice were sacrificed and lungs were examined for metastatic lesions (number and size) under stereoscopic microscope (Bausch & Lomb).

### Adhesion assay

RGZ (1 μM) was added to LMM3 cells during the process of adhesion to 6-well plates in the absence of FCS. After 2 h, the number of non-adherent cells was determined by counting cells in the supernatants ("process of adhesion assay"). In another set of experiments, RGZ was added for 6h to already attached cells and the number of cells that could be obtained by aspiration was determined. ("resistance to aspiration assay"). When used, 10 μM BADGE was added 15 min before RGZ.

### Migration Assay

A wound of 400 μm was made on confluent cell monolayers in 6-well plates and 10 μM RGZ with or without BADGE was added ("wound assay"). Microphotographs taken immediately after performing the wound (T0) and 24h later (T1) were analyzed using the Image-ProPlus 4.5 software program. Difference between cell-free area at T0 and T1 was quantified.

### Invasion assay

50 μl 0.1% gelatine and 100 μl matrigel were loaded in the lower and upper part respectively of Transwell chambers. Using fibronectin (4 μg/ml) as chemoattractant 2 × 10^5 ^LMM3 were seeded and treated with 1 μM RGZ, 10 μM BADGE or the combination of both. After 20 h culture at 37°C, membranes were fixed and stained with DAPI. Cell invasion was determined counting the number of cells adhered to the membranes using a fluorescence microscope (400× magnification). 10 fields were counted per group.

### Gelatin zymography

Gelatinolytic MMP activity was determined on substrate-impregnated gels. Briefly, samples were separated on SDS-9% polyacrylamide gels containing 1 mg/ml copolymerized gelatin (Difco, Detroit, MI) under non-reducing conditions. These gels were washed twice with 2.5% Triton X-100, incubated for 24 hours at 37°C in 0.25 M Tris-HCl, 1 M NaCl, 25 mM CaCl_2 _at pH 7.4, and stained with 0.5% Coomassie G 250 (Bio-Rad, Richmond, CA) in methanol/acetic acid/water (30:10:60). The clear lysed areas (white bands) on the stained gels were measured with a GS-700 densitometer (Bio-Rad, Hercules, CA, USA).

### Immunohistochemistry

PPARγ expression was analyzed in formalin-fixed, paraffin-embedded sections of LMM3 tumors. Briefly, endogenous peroxidase activity was blocked with 0.6 % hydrogen peroxide. Antigen retrieval was performed using microwave oven irradiation in citrate buffer (pH: 6). Slides were incubated for 1 h with normal goat serum (1:10) (Vector Labs, Burlingame, CA) and overnight with rabbit specific monoclonal antibody anti-PPARγ (H-100, Santa Cruz Biotechnology, USA) diluted 1:20. Sections were incubated with biotinylated goat anti-rabbit IgG (Sigma-Aldrich) diluted 1:500 for 60 min, followed by incubation with peroxidase-conjugated streptavidin diluted 1:3000 in phosphate-buffered saline for 45 min. Peroxidase reaction was performed using 0.02% 3, 3'-diaminobenzidine tetrahydrochloride and 0.01% hydrogen peroxide and co-staining with hematoxylin (1 min). As negative control, the primary antibody was omitted.

### Detection of PPARγ by western blot

LMM3 cell monolayers were rinsed twice with ice-cold PBS and scraped into 100 ul RIPA lysis buffer: (50 mM Tris-HCl, pH 7.5; 150 mM NaCl, 10 mM EDTA, 1% NP40). Lysis was completed by sonication and centrifugation. Protein content was determined with Bradford reagent, using bovine serum albumin as standard. Sample electrophoresis (100 μg protein/25 μl/lane) was performed on 10 % sodium dodecyl sulfate polyacrylamide gels (SDS-PAGE). Proteins were transferred to nitrocellulose membranes and incubated overnight with mouse monoclonal antibody anti-PPARγ, diluted 1:100. The secondary antibody, conjugated with alkaline phosphatase anti-rabbit IgG (Sigma-Aldrich), was added for 1 h at 37°C. Proteins were visualized using a mixture of NBT/BCPI (Sigma-Aldrich) and scanned with a computerized densitometer (GS-700Calibrated Densitometer Bio-Rad Laboratories, Richmond, CA).

### Statistical analysis

Results are expressed as mean ± standard deviation. Significance of differences was calculated by Student's t test or Mann Whitney. All experiments were performed by triplicate and repeated at least three times, with comparable results.

## Results

### RGZ and 15d-PGJ2 differentially affect LMM3 cell viability and NO production

**Figure 1:**
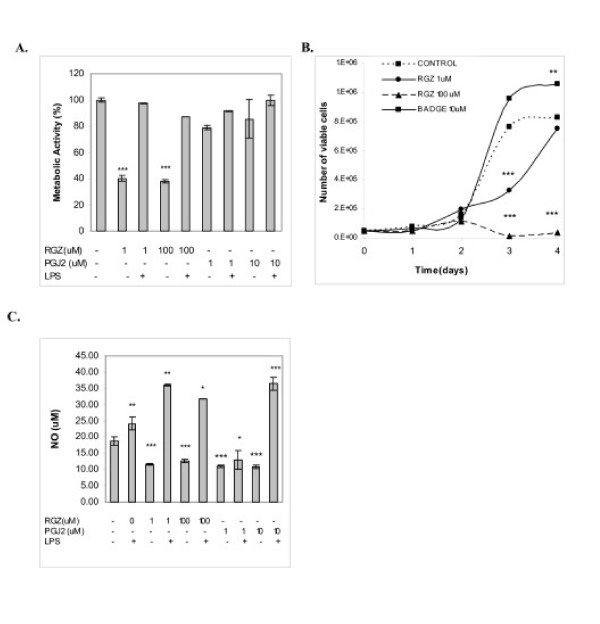
**Effect of RGZ and 15d-PGJ_2 _on LMM3 cell viability**. **A**. MTS assay was used to determine cell metabolic activity after 24 h-culture in the presence of RGZ (1 and 100 μM) and 15d-PGJ_2 _(1 and 10 uM) with or without LPS (500 μg/mL). Results are expressed as absorbance (% of control considered 100%) ***p < 0.001 vs. control. **B**. The growth-kinetics of LMM3 cells in the presence of 10 μM BADGE, 1 and 100 μMRGZ is shown (**p < 0.01, ***p < 0.001 vs. control). **C**. NO production by LMM3 tumor cells cultured during 24 h with RGZ (1 and 100 μM) or 15d-PGJ_2 _(1 and 10 μM) was measured in the supernatants using Griess reagent. Experiments were performed with and without LPS. Results are expressed as μM NO_2 _respect to a control curve of nitrite. A and B show the results of one representative experiment of three independent assays performed by triplicate with similar results. **p < 0.01; ***p < 0.001 vs control.

1 and 100 μM RGZ reduced metabolic activity in 50%, measured with MTS assay (Fig 1A). To determine if this inhibition was associated with a decrease in cell viability, the number of viable cells was quantified every 24 h up to 96 h (Trypan blue exclusion test). After 72 h, control cells reached a plateau and the number of viable cells quadrupled the number of seeded cells. In cells cultured with 1 μM RGZ, no significant increase in cell number was observed during the first 48 h. At this point cells began to recover their proliferating capacity, and reached control numbers at 96 h. On the contrary, cells treated with 100 uM RGZ almost died after 72 h (Fig 1B). 15d-PGJ_2 _did not affect metabolic activity.

Since inflammation is an important component of tumor microenvironment, we examined the effect RGZ in cell cultures with LPS. Addition of LPS completely abrogated the inhibitory effect of 1–100 μM RGZ (Fig 1A). Previous results have shown that NO seems to be a protective/survival factor for LMM3 cells, since increased cell death was detected in the presence of NOS inhibitors such as L-NAME (N^G^-nitro-L-arginine methyl esther) and AG (aminoguanidine) (Jasnis MA et al, unpublished results). NO production decreased 40% in cultures with 10 μM15d-PGJ_2 _and 1–100 μM RGZ; the presence of LPS reversed this effect and induced a significant increase in NO levels (Fig 1C).

### Effect of BADGE and GW9662 on cell viability

**Figure 2:**
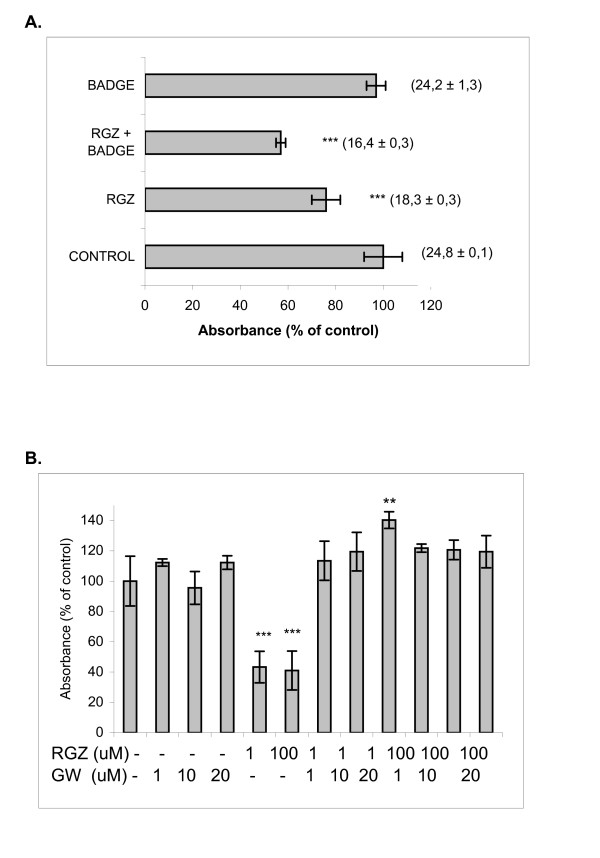
**A. Effect of BADGE on cell metabolic activity**. BADGE (10 μM) was added to cell cultures alone or 15 minutes before 100 μM RGZ. After 24 h, metabolic activity was determined by MTS assay. BADGE *per se *had no effect. BADGE + RGZ diminished metabolic activity compared to RGZ alone (*** p < 0.001). NO levels are shown between parentheses (μM). B. Effect of GW-9662 on LMM3 cells. GW-9662 (1, 10, and 20 μM) was added to cell cultures 1 h before 100 μM RGZ. After 24 h, metabolic activity was determined by MTS assay. (*** p < 0.001 vs. control untreated cultures).

Since BADGE and GW9662  are PPARγ antagonists, we studied their effects to assess the relative roles for PPARγ-dependent and independent biological responses. 10 μM BADGE and GW9662 (1, 10 and 20 μM) *per ser *did not affect cell metabolic activity while higher concentrations of BADGE were cytotoxic (data not shown). As shown in Fig 2a, BADGE did not behave as an antagonist. Moreover, a more significant decrease in metabolic activity with BADGE + RGZ was detected compared to RGZ alone. The same effects were observed on NO production (Fig 2a, numbers between parentheses). On the other hand, GW-9662, an irreversible PPARγ antagonist, completely reversed the inhibition of cell metabolic activity induced by RGZ 1 and 100 μM. (Fig 2b)

#### Tumor and metastasis growth

**Figure 3:**
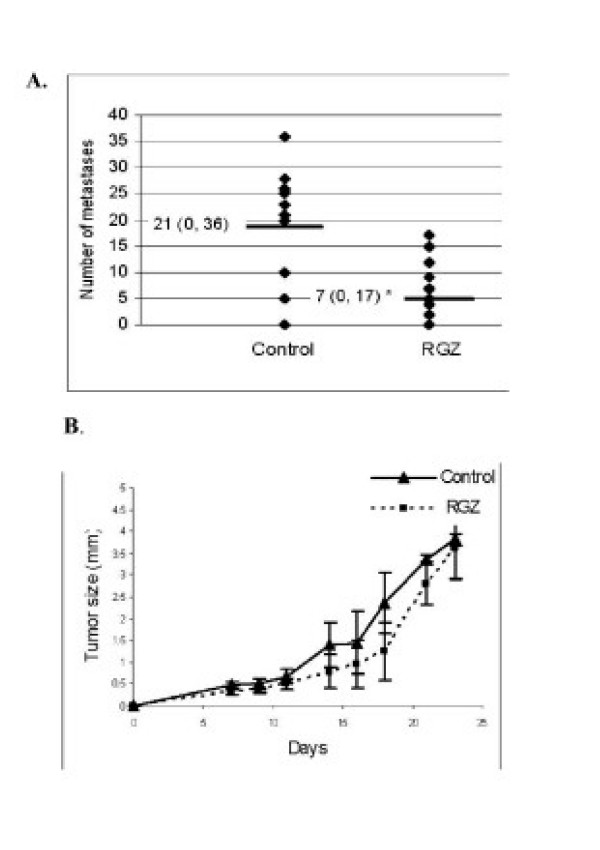
**Effect of RGZ on experimental lung metastases and primary tumor growth**. **A**. Lung metastasis. On day 0, mice (n = 10/group) were i.v. inoculated with LMM3 cells into the tail vein. Experimental group was orally treated with 100 μMRGZ while control group only with ethanol in drinking water. On day 30, mice were sacrificed and number and size of lung metastatic nodules were recorded under microscope. Each point in the figure represents total number of metastases/lung/mice. All nodules were ≤ 2 mm in diameter. Median (range) is shown on the horizontal line (p < 0.007). One experiment of three with similar results is shown. **B**. Primary tumor. Perpendicular diameters of s.c. growing tumors were measured three times/week since day 7 (latency) up to day 25. Average of tumor size at each point was calculated as follows: √(dxD), d = minor diameter, D = major diameter. Error bars represent SD (n = 10). No significant difference was observed at any time during tumor progression between groups.

Tumor cells were directly inoculated into the tail vein and differences in lung metastasis were detected between untreated and RGZ-treated mice: median (range): 21 (5–36) and 7 (0–17) respectively, p < 0.0001 (Fig 3A). These differences were observed in metastasis with diameters up to 2 mm. However, oral administration of RGZ did not affect primary s.c. tumor growth (Fig 3B).

#### Effect of RGZ on cell adhesion and migration

**Figure 4:**
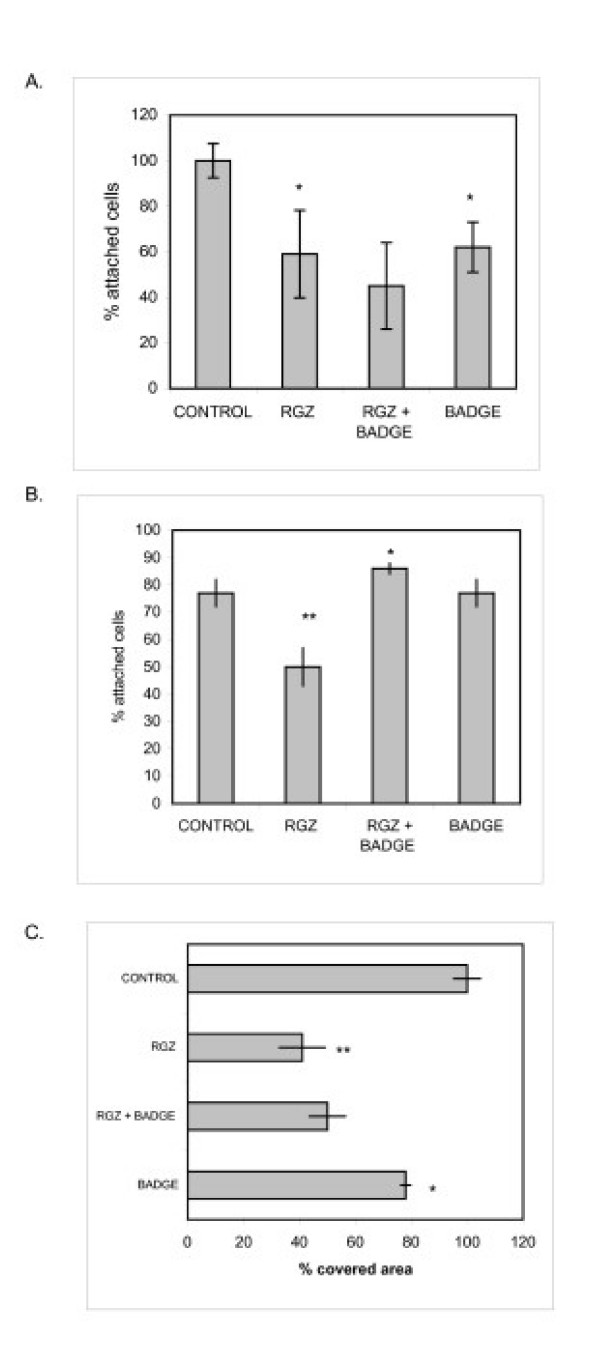
**Effect of RGZ on cell adhesion and migration**. **A**. LMM3 cells were cultured with 1 μMRGZ and 10 μM BADGE alone or in combination. Non-adherent cells were counted after 2 h with a haemocytometer. * p < 0.05 vs. control. No differences were observed between RGZ and RGZ+BADGE. **B**. Already attached tumor cells were treated with RGZ with or without BADGE. The number of detached cells after 8 h was considered as a measure of "resistance to aspiration" ** p < 0.01 vs. control; * p < 0.05 vs. RGZ; **C**. Cell migration in the presence of 1 μM RGZ, 10 μM BADGE or the combination of both was evaluated using the "wound assay". The % of covered area after 24 h treatment, compared to control is shown. In all cases representative results of three experiments performed by triplicate are shown.

During early cell attachment (2 h), 1 μM RGZ significantly inhibited tumor adhesion. The addition of BADGE did not reverse this effect, and BADGE *per se *diminished cell adhesion (Fig 4A). After 8 h adhesion 77% of control cells were resistant to aspiration. Addition of RGZ diminished by 50% cell attachment  (50% attached cells) (Fig 4B). It is noteworthy that BADGE could reverse the inhibitory effect completely (Fig 4B). A reduction of 59% in tumor cell migration was observed in the presence of RGZ. This result was not reversed by BADGE -which slightly inhibited cell migration *per se*. (Fig 4C).

#### Cell Invasion in Matrigel

**Figure 5:**
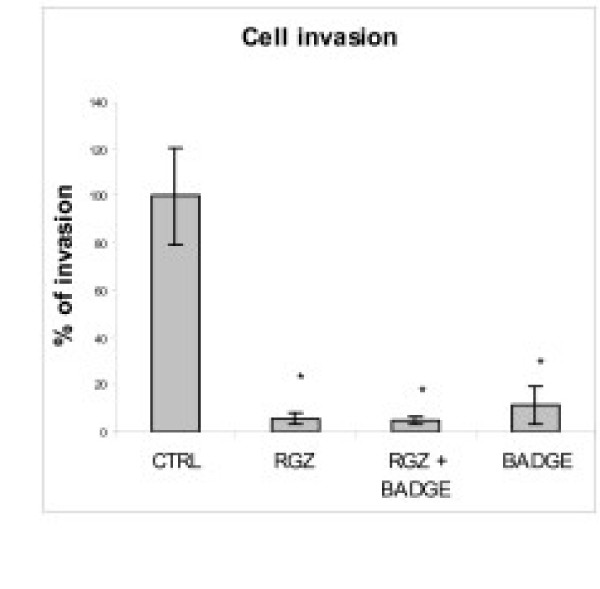
**Effect on Cell Invasion in Matrigel**. Inhibition in cell invasion in matrigel was detected in all cultures, with no differences between 1 μM RGZ and 10 μM BADGE alone or in combination (*p < 0.05).

RGZ and BADGE at non-toxic doses, alone or in combination, completely annulled LMM3 cell invasion in Matrigel (Fig 5).

#### Production of matrix metalloproteases

**Figure 6:**
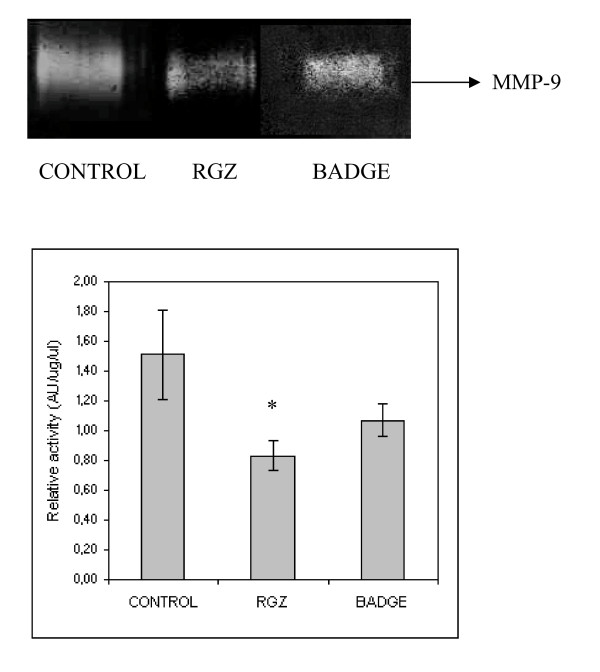
**Effects on MMP activity**. MMP activity was determined in conditioned media from control LMM3 tumor cells (left), 1uMRGZ-treated (middle) and 10 μM BADGE-treated cells (right). RGZ induced a decrease on the expression of latent MMP-9 (LMMP-9) compared to control (*p < 0.05).

MMP activity was analyzed by gelatin and casein zymography in conditioned media of control, 1 µM RGZ and 10 µM BADGE treated LMM3 cells. Gelatin zymography revealed bands corresponding to mouse mammary gland latent MMP-9 (LMMP-9). A decrease in the levels of LMMP-9 was detected in conditioned media from RGZ and BADGE treated cells compared to control untreated cells. (Fig. 6)

#### Effects of RGZ on PPARγ expression

**Figure 7:**
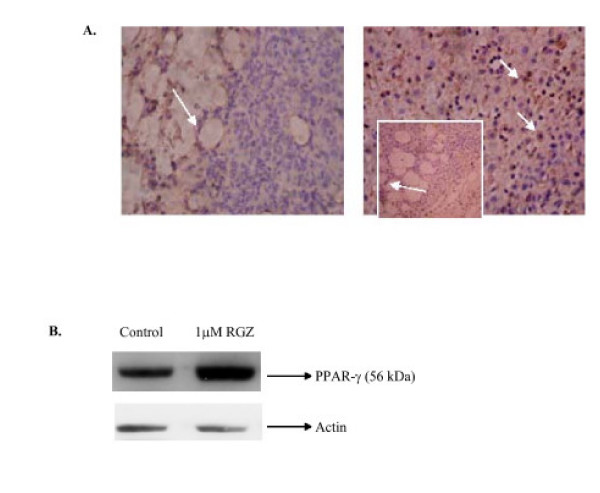
**A. PPARγ expression in LMM3**. Representative immunostaining for PPARγ in LMM3 cells from fresh tumor samples obtained from untreated mice (right panel) and from RGZ-orally treated (100 μM) (left panel). Almost all tumor cells were positive in tumors from RGZ treated animals while no staining was detected in LMM3 cells from tumors from untreated animals. Adipocytes and endothelial cells were positive in both groups. Photograph magnification, ×400. **B**. Western blot and immunostaining of PPARγ. Cell lysates were prepared from LMM3 tumor cells treated or not with 1 μM RGZ for 24 h. Western blot analysis was performed with specific antibody anti PPARγ.

Perinuclear staining of PPARγ was detected in almost all tumor cells from RGZ-treated mice (Fig 7 right panel), while no expression could be found in tumors from untreated mice (Fig 7 left panel). In treated and untreated tumor bearing mice, adipocytes and endothelium showed intense PPARγ staining. RGZ enhanced the expression of PPARγ protein in LMM3 cells, compared to control cells (Western blot, Fig. 7B).

## Discussion

The main finding of our experiments is the decrease in the number of experimental LMM3 lung metastasis after oral RGZ administration without any effect on primary tumor growth. Metastasis inhibition could be correlated with *in vitro *results of metabolic activity and cell viability reduction by 100 μMRGZ. 1 μM RGZ only induced the decrease in metabolic activity without affecting cell viability, suggesting a possible arrest in cell cycle. It is probable that oral ingestion of a solution 100µM does not reach this same plasmatic concentration, but most likely achievable doses obtained *in vivo* are closer to 1µM., partially explaining the absence of *in vivo* tumor killing. 10 μM BADGE *per se *significantly enhanced the number of viable cells over control cultures, showing that at non-toxic doses, it was able to stimulate cell proliferation. Moreover, BADGE not only did not reverse the inhibitory effect of RGZ, but instead acted to further decrease cell metabolism (Fig 2A). Our results agree with those of Lea et al [14] who reported that BADGE and diclofenac failed to reverse the inhibitory effect of ciglitazone on growth of human breast cancer and mouse eritroleukemia cells. Although it has been widely reported that doses near to 100 uM are needed for BADGE to behave as a PPAR inhibitor, we could not use higher doses than 10 uM because, in our system, they were cytotoxic. BADGE has been shown to serve as a PPARγ agonist in RAW 264.7 cells, human monocytes and in some epithelial cells [15,16] and as an inducer of apoptosis in some tumor cells [17]. The mechanisms responsible for the effects of RGZ on LMM3 viability seem to involve PPARγ-dependent signals since we found that changes in cell metabolism induced by RGZ were inhibited by GW9662. Our results agree with those reported using different NSCLC cell lines [11]. Studies with a great variety of solid and hematological human tumors have demonstrated the antitumor effects of different TZDs [18-20]. It has also been reported that RGZ slowed proliferation but did not induce apoptosis of colon cancer cells [21]. In our model, inhibition of metabolic activity with 1 uM RGZ was not associated with cell death but most likely with a reversible cell cycle arrest, since after 72 h, cultured cells recovered proliferating capacity. Morosetti et al have found that after drug removal, cells exposed to RGZ resumed their proliferation, while cells treated with PGJ_2 _did not overcome the cytotoxic insult [22]. These differences were associated with an irreversible G2/M arrest induced by15d-PGJ_2 _and only transiently by RGZ. LMM3 cell viability was not affected by 15d-PGJ_2_. The differences between natural and synthetic ligands can be attributed to distinctive affinities or potency of both ligands in LMM3 cells. RGZ and 15d-PGJ_2 _have been shown to exert anti-proliferative effects on human glioblastoma cell lines but only PGJ_2 _modulated the expression of proteins associated with cell differentiation [22] Differences in the level of PPARγ may also affect the overall sensitivity of a tumor cell to activating ligands. The MCF-7 cell line, which expresses rather low amounts of PPARγ in comparison to other cell lines, was sensitive only at high concentration of PGJs [23] Receptor mutations leading to altered binding affinities or activating effects could also account for differences on sensitivity [24].

Inhibition of LMM3 lung metastatic nodules by RGZ might be associated with the observed decrease in MMP-9 expression. Opposite results have been recently reported demonstrating that, in the HT1080 cell line, pro-MMP-2 was activated by ciglitazone and that the antagonist GW9662, although attenuated PPARγ activation, had no effect on MMP-2 [25]. Cell adhesion and adhesion molecules are critical in the processes of invasion and metastasis. The anti-adhesive properties of RGZ were observed both during the process of LMM3 cell adhesion and during de-adhesion of already attached cells. It is interesting to note that in early adhesion, BADGE and RGZ showed the same anti-adhesive effect. However, once the cells were already attached, addition of BADGE reversed RGZ-induced cell detachment. Combination of RGZ and BADGE showed that BADGE is not a pure antagonist of PPARγ since, in the process of early adhesion; it seemed to act as an agonist ligand. It is noteworthy that loss of LMM3 cell adhesion was not the result of cell death since all detached cells were still alive. It has been reported that PPARγ inhibitors prevented thyroid and hepatocellular carcinoma cell adhesion by inhibition of FAK phosphorylation and inducing cell death by anoikis [26,27]. Furthermore, in a model of colorectal cancer, it was recently observed that BADGE was a potent inhibitor of metastases [28]. It is becoming clear that activation of the same receptor with different ligands may result in different responses. The anti-metastatic activity of RGZ in vivo, without affecting primary tumor growth, might be partially attributed to inhibition of cell adhesion, migration ("wound assay") and invasion (Transwell), the latter being the most sensitive.

The role of PPARγ in tumors has been widely studied. PPARγ agonists induce apoptosis using fatty acid derivatives, TZDs and tyrosine-based agonists in several cancer cell types [29]. More recently, perturbation of PPARγ expression and activity have been suggested as a therapeutic strategy for several epithelial tumor types [30]. Studies on the expression of PPARγ in LMM3 tumor cells showed that RGZ augmented its expression. PPARγ was detected by immunohistochemistry only in tumors from RGZ-treated mice, while surrounding adipocytes and endothelium were stained in treated and control animals. According to these observations, we cannot discard the fact that *in vivo*, the inflammatory tumor microenvironment (macrophages, lymphocytes, endothelial cells) might regulate PPARγ expression in tumor cells. It has been reported that serum lysophosphatidic acid attenuates both the degree of PPARγ activation and the cellular response to 15d-PGJ_2 _in neuroblastoma cells [31].

The decrease in NO levels in the presence of RGZ was dose-independent. LMM3 cells express COX-1, COX-2, NOS2, NOS3 and arginase II, involved in angiogenesis and tumor cell migration. We provided evidence that PGE_2 _exerted a positive loop on NOS activity in these tumor cells, augmenting NO levels which closed the loop with a negative feed-back on COX activity [32]. Previoulsy, we have reported that LMM3 cells produce high levels of NO, further increased in the presence of LPS+IFN, which inversely correlated with sensitivity to injury by an exogenous source of NO [33]. In the present experiments, both RGZ and 15d-PGJ_2 _reduced NO production, a survival factor for LMM3 cells (Jasnis MA et al, unpublished data). In other tumor model, we have observed that induction of PPARγ expression by BCG, 15d-PGJ_2 _and RGZ was associated with a decrease in NO production and loss of cell viability [34].

Since inflammation is an important component of tumor progression, we analyzed the effect of an inflammatory stimulus such as LPS on the response of LMM3 cells to RGZ or 15d-PGJ_2_. While NO production was augmented by RGZ, it decreased by 15d-PGJ_2_. Furthermore, LPS reversed cell metabolic inhibition induced by RGZ. It has been reported that LPS suppressed PPARγ expression in human monocytes [35] and in microglial cultures [36]. Agonists of PPARγ are also able to modulate inflammatory responses in several cells by inhibiting the expression of proinflammatory cytokines and of inducible NOS and COX-2 [5,37]. However, other studies have yielded opposite results showing that RGZ increased the inflammatory response in epithelial cells and in macrophages [38,39].

## Conclusion

The current study suggests that RGZ or any other TZDs should be considered as possible future therapeutics in the treatment of metastasis of PPARγ-expressing cells. Since we cannot underestimate the influence of the inflammatory milieu of the tumor microenvironment, it will be necessary to understand the mechanism of action in a more profound way, and to define under which circumstances these compounds work best *in vivo*.

## Competing interests

The author(s) declare that they have no competing interests.

## Authors' contributions

GM performed the bulk of experiments in vitro and in vivo as part of PhD Fellowship from ANPCYT and participated in the drafted of the manuscript. XB participated in the in vitro experiments with RR. MAJ conceived the study and participated in its design and coordination. All authors read and approved the manuscript.

## Pre-publication history

The pre-publication history for this paper can be accessed here:


